# Case Report: AFP-producing gastric hepatoid adenocarcinoma with multiple liver metastases – integrating quantitative imaging and diagnostic decision analysis

**DOI:** 10.3389/fonc.2026.1768282

**Published:** 2026-03-25

**Authors:** Shi-Yu Zhong, Xiang-Rong Deng, Yun-Jiao Zhou, Ting Liu, Xiao-Yu Xu, Xiang-Ke Niu, Jian-Ming Xiao, Tao Peng

**Affiliations:** 1 Department of Radiology, Chongzhou Hospital of Traditional Chinese Medicine, Chongzhou/Chengdu, Sichuan, China; 2 Clinical Medical College of Southwest Medical University, Luzhou, Sichuan, China; 3 Department of Radiology, Affiliated Hospital of Zunyi Medical University, Zunyi, Guizhou, China; 4 Department of Radiology, Affiliated Hospital of Chengdu University, Chengdu, Sichuan, China

**Keywords:** alpha-fetoprotein (AFP), computed tomography (CT), differential diagnosis, gastric cancer, hepatoid adenocarcinoma, liver metastasis

## Abstract

**Background:**

Hepatoid adenocarcinoma of the stomach (HAS) is a rare and aggressive gastric cancer variant with both glandular and hepatocellular differentiation. The AFP-producing subtype is strongly associated with early liver metastasis and poor prognosis, presenting significant diagnostic challenges due to its resemblance to hepatocellular carcinoma (HCC).

**Case summary:**

A 68-year-old male presented with progressive dizziness and fatigue. Initial contrast-enhanced abdominal CT revealed a hypervascular gastric antral mass with quantitative enhancement values of 52 ± 3 HU (arterial), 78 ± 5 HU (portal), and 85 ± 4 HU (delayed) in the submucosal layer. Serum AFP was markedly elevated at 918.88 ng/mL. Gastroscopic biopsy confirmed AFP-producing HAS. The patient underwent Billroth II gastrectomy. Four months postoperatively, surveillance CT identified multiple new hypervascular liver metastases, prompting right hepatectomy followed by adjuvant radiochemotherapy. At three-month follow-up, the patient was asymptomatic with normalized tumor markers.

**Conclusion:**

This case illustrates the utility of quantitative CT enhancement analysis for characterizing the hypervascular phenotype of AFP-producing HAS and proposes a conceptual diagnostic framework to differentiate HAS from HCC. Multidisciplinary collaboration and systematic serum AFP screening in patients with gastric lesions are essential for optimal management of this aggressive malignancy.

## 1 Introduction

Hepatoid adenocarcinoma of the stomach (HAS), first conceptualized by Ishikura et al. (1) in 1985, is a distinct clinicopathological entity recognized in the WHO classification (2), characterized by hepatocellular differentiation and frequent alpha-fetoprotein (AFP) production. This rare subtype, accounting for only 0.17%-0.73% of gastric cancers, exhibits markedly aggressive biological behavior compared to conventional adenocarcinoma. Notably, it demonstrates a strikingly high rate of liver metastasis (up to 75.6%) and a correspondingly poor 5-year survival rate of approximately 9% (3). The frequent elevation of serum AFP, a hallmark feature, often leads to diagnostic confusion with primary hepatocellular carcinoma (HCC), potentially delaying correct management (4–6). This report presents a case of AFP-producing HAS with metachronous liver metastases and incorporates quantitative imaging analysis to provide a practical diagnostic perspective. This integrated approach aims to enhance diagnostic accuracy and therapeutic planning for this challenging entity.

## 2 Case description

### 2.1 Clinical presentation and initial work-up

A 68-year-old male with a 2-year history of iron-deficiency anemia presented to the emergency department with exacerbating dizziness, profound weakness, and a recent syncopal episode. The patient was initially admitted to the hematology department for investigation of anemia of unknown origin, given his symptoms and positive fecal occult blood test. During this evaluation, a contrast-enhanced abdominal CT scan was performed, which incidentally revealed a suspicious gastric antral mass. Consequently, he was referred to the gastroenterology department for further diagnostic work-up. His past medical history was significant for hypertension, coronary artery disease status post stenting and pacemaker implantation, and chronic bronchitis. Physical examination revealed pallor and signs of chronic anemia; no other abnormalities were noted. Initial laboratory investigations revealed a significantly elevated serum AFP level of 918.88 ng/mL (institutional reference range: <7.4 ng/mL) and positive fecal occult blood.

### 2.2 Imaging findings (initial staging)

A multiphasic contrast-enhanced abdominal CT scan was performed using a standardized protocol (120 kVp, automatic tube current modulation, 1.5 mm slice thickness). Imaging analysis included both qualitative assessment and semi-quantitative measurements:

• Primary Gastric Lesion: A circumferential, irregularly thickened wall of the gastric antrum/pylorus, measuring up to 1.9 cm in maximum thickness (normal gastric wall thickness is typically <5 mm). On dynamic enhancement, the lesion exhibited a hypervascular phenotype: mild arterial phase enhancement followed by pronounced progressive enhancement in the portal venous and delayed phases.

For quantitative analysis, three regions of interest (ROIs, diameter ≈5 mm) were placed within representative layers of the tumor. The mean enhancement values (± standard deviation) in Hounsfield Units (HU) were as follows:

Submucosal region: Arterial phase: 52 ± 3 HU; Portal phase: 78 ± 5 HU; Delayed phase: 85 ± 4 HU.
Muscular region: Arterial phase: 42 ± 4 HU; Portal phase: 65 ± 3 HU; Delayed phase: 72 ± 5 HU.
Serosal region: Arterial phase: 38 ± 2 HU; Portal phase: 60 ± 4 HU; Delayed phase: 68 ± 3 HU.

**Figure 1:**
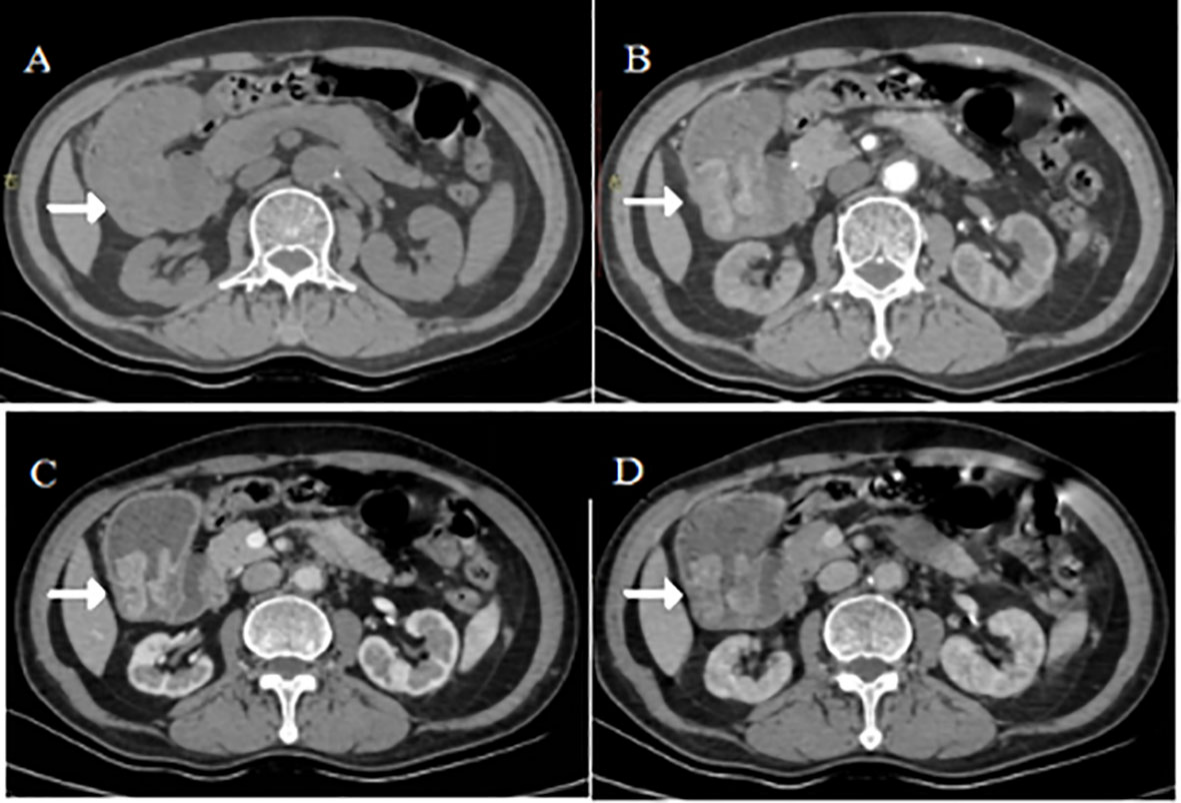
Initial staging CT (February 2020). Axial contrast-enhanced CT images in **(A)** arterial, **(B)** portal venous, and **(C)** delayed phases, with **(D)** coronal reformat, demonstrate an irregularly thickened, hyperenhancing gastric antral wall (white arrows), characteristic of the primary hepatoid adenocarcinoma.

The submucosal region demonstrated the most pronounced enhancement, with a pattern resembling the hemodynamics of hepatocellular carcinoma (“rapid-in with sustained enhancement”) (**Figures 1A–D**). This radiographic phenotype correlated with the hepatoid differentiation observed on subsequent histopathology.

Liver and Lymph Nodes: No evidence of hepatic metastasis or significantly enlarged abdominal lymph nodes was identified on this initial scan.

### 2.3 Pathological diagnosis and immunohistochemistry

**Figure 2:**
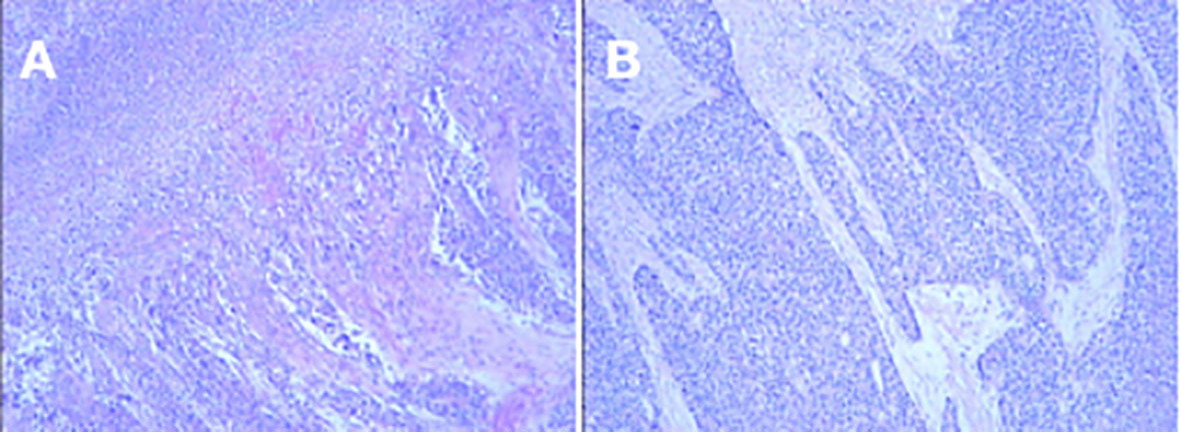
Histopathology of primary gastric tumor (H&E, 40x). **(A)** Shows a composite morphology with areas of conventional adenocarcinoma (lower left) adjacent to solid sheets of hepatoid cells with abundant cytoplasm (upper right). **(B)** Highlights the presence of vascular tumor thrombus (arrow), indicative of angiolymphatic invasion.

Upper gastrointestinal endoscopy revealed an ulcerated, bleeding mass in the gastric antrum. Histopathological examination of biopsy specimens showed a poorly differentiated carcinoma with biphasic morphology: conventional gland-forming adenocarcinoma adjacent to solid sheets of polygonal cells with abundant eosinophilic cytoplasm resembling hepatocytes (**Figure 2A**).

Immunohistochemical (IHC) profiling was diagnostic:

- Positive: AFP (strong, diffuse), HepPar-1 (cytoplasmic), CD34 (vascular staining), CK7 (focal), SMA (highlighting tumor vasculature).
- Negative: CD10, CD117, CK20.
- Proliferation index: Ki-67 approximately 60%.

These findings confirmed the diagnosis of AFP-producing hepatoid adenocarcinoma of the stomach. This immunoprofile (AFP and HepPar-1 positivity) is characteristic of HAS and is employed both for diagnosis and prognostication (7, 8).

### 2.4 Treatment and follow-up imaging

The patient underwent curative-intent Billroth II gastrectomy with D2 lymphadenectomy. Final pathological examination confirmed a 5.5 × 5.0 × 1.0 cm tumor invading the muscularis propria with lymphovascular invasion and metastasis in 2 of 17 regional lymph nodes.

**Figure 3:**
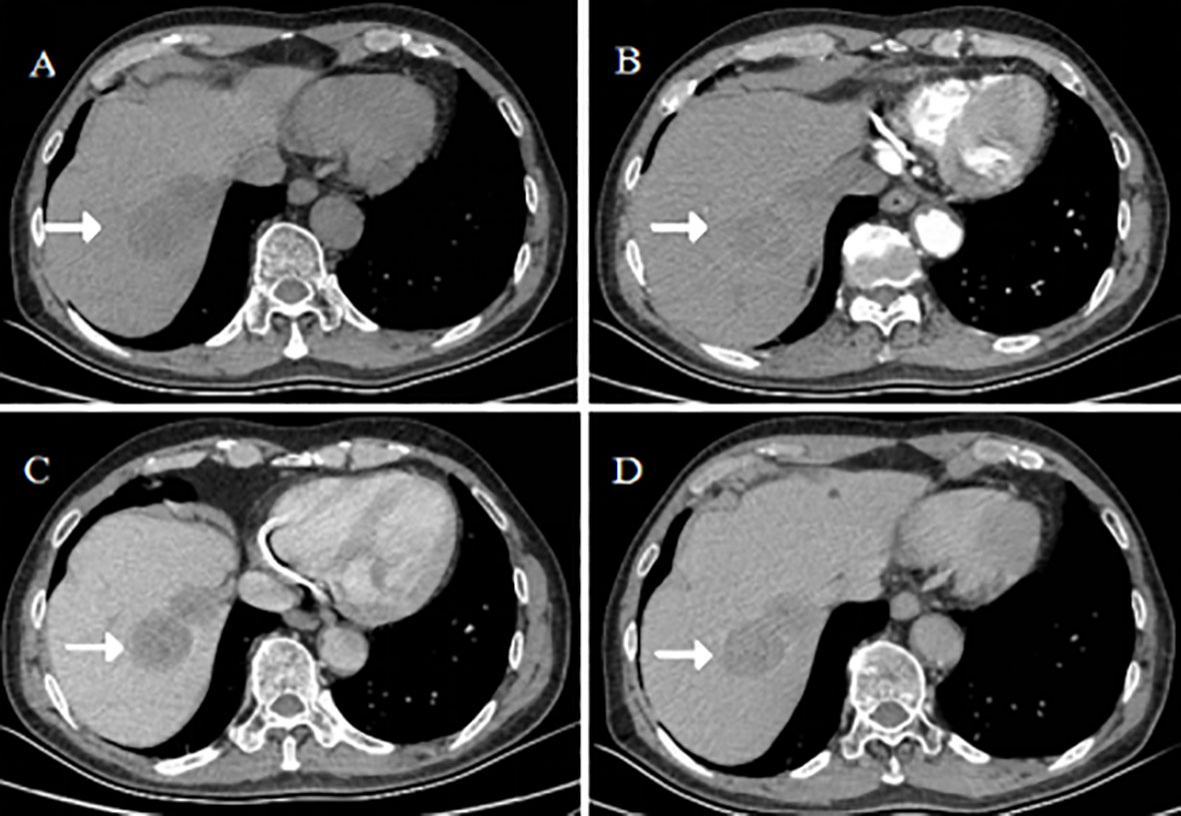
Surveillance CT detecting liver metastases (February 2021). Axial post-contrast CT images in **(A)** arterial and **(B)** portal venous phases reveal new, rim-enhancing metastatic lesions in the right hepatic lobe (white arrows). The largest metastasis in segment VII is shown in **(C)** axial and **(D)** coronal planes.

Four months postoperatively, surveillance contrast-enhanced CT revealed multiple new hypodense lesions in the right hepatic lobe. The largest lesion (segment VII) exhibited characteristic peripheral rim enhancement on arterial phase with progressive centripetal fill-in, consistent with hypervascular metastases (**Figure 3**). After multidisciplinary discussion, the patient underwent right hemihepatectomy. Histopathology confirmed metastatic hepatoid adenocarcinoma, immunophenotypically identical to the primary gastric tumor. The patient subsequently received adjuvant chemoradiotherapy (capecitabine with concurrent radiotherapy) and tolerated it well.

The chronology of care is summarized below (see also **Supplementary Table 1** for a detailed timeline):

- Initial Presentation (Feb 2020): Symptoms, elevated AFP, CT reveals gastric mass.
- Feb 2020: Gastroscopic biopsy confirms HAS.
- Mar 2020: Billroth II gastrectomy.
- Feb 2021 (4 months post-op): Surveillance CT detects liver metastases.
- Mar 2021: Right hemihepatectomy.
- Apr 2021 onward: Adjuvant chemoradiotherapy.
- At the three-month follow-up after liver resection, the patient remained clinically asymptomatic with normalized serum AFP (8.2 ng/mL) and no imaging evidence of recurrence.

### 2.5 Patient perspective

The patient was informed of the rarity and aggressive nature of his diagnosis. He expressed understanding of the need for sequential surgical interventions (gastrectomy and hepatectomy) and subsequent adjuvant therapy. During follow-up, he reported satisfaction with the multidisciplinary care provided and relief at the normalization of his tumor marker levels and absence of symptoms. He provided consent for the publication of his case to contribute to medical knowledge.

## 3 Diagnostic assessment and therapeutic intervention

### 3.1 Diagnostic assessment

The diagnosis rested on a triad of findings: 1) markedly elevated serum AFP, 2) a hypervascular gastric mass with a quantitative enhancement profile suggestive of hepatoid differentiation, and 3) definitive histopathological and IHC confirmation of biphasic tumor morphology with hepatocytic markers (AFP, HepPar-1).

Diagnostic methods included multiphasic CT (section 2.2), upper endoscopy with biopsy, and immunohistochemistry (section 2.3).

Diagnostic challenges: The main challenge was distinguishing HAS from HCC, given the overlapping features of hypervascularity and AFP production. This was addressed by recognizing the gastric origin and the absence of underlying liver disease. As noted in section 4.2, hepatoid morphology and AFP production are not specific to gastric origin, with reported cases in diverse sites (9–14).

Differential diagnosis: Key differentiators between HAS and HCC are summarized in **Table 1**.

**Table 1:** Key differentiators between hepatoid adenocarcinoma of the stomach (HAS) and hepatocellular carcinoma (HCC).

Differentiator	Hepatoid adenocarcinoma of the stomach (HAS)	Hepatocellular carcinoma (HCC)
Primary Site	Dominant gastric wall mass/thickening, often ulcerated. Liver lesions are metastatic.	Dominant liver mass. Gastric involvement, if any, is exceedingly rare and typically by direct serosal invasion from adjacent liver.
Clinical Background	Usually no underlying chronic liver disease or cirrhosis.	Strong association with chronic hepatitis B/C infection, liver cirrhosis, or other chronic liver diseases.
Pattern of Liver Involvement	Multiple, bilateral metastases are common at presentation. May exhibit “rim enhancement”.	Often solitary or a few nodules within a cirrhotic liver. Classic enhancement is “wash-in and washout”.
Lymph Node Metastasis	Frequent regional (perigastric) and abdominal lymph node metastasis.	Less common at initial presentation; when present, often indicates advanced disease.
Histology & IHC Profile	Biphasic morphology (adenocarcinoma + hepatoid areas). IHC: AFP+, HepPar-1+, CK7+ (focal), CK19+, CD34 (stromal+). Often negative for CD10 (canalicular pattern).	Pure hepatoid morphology. IHC: AFP+, HepPar-1+, CD10 (canalicular pattern+), Glypican-3+. Typically negative for CK7 and CK19.\

Prognosis: Based on literature, HAS has a high risk of liver metastasis and poor 5-year survival (3). In this patient, pathological stage (T2 N1 M0 at presentation) and subsequent metachronous metastases confirm the aggressive nature, warranting intensive surveillance.

### 3.2 Therapeutic intervention

The therapeutic strategy was predicated on achieving complete (R0) resection of both primary and metastatic disease, given its potential for cure in oligometastatic settings. This was followed by adjuvant therapy, extrapolated from aggressive gastric adenocarcinoma protocols due to the lack of HAS-specific guidelines, to address the high risk of micrometastatic disease.

## 4 Discussion

### 4.1 Quantitative imaging phenotype and pathological correlation

As detailed in the Case Description (2.2), the quantitative CT analysis serves as an example of how routine measurements can support diagnosis. The pronounced enhancement in the submucosal tumor region (arterial: 52 ± 3 HU; portal: 78 ± 5 HU; delayed: 85 ± 4 HU) correlates with the histopathological finding of hepatoid differentiation in this area, which typically features rich sinusoidal vasculature reminiscent of hepatic parenchyma. This “hepatoid enhancement pattern” - rapid arterial uptake with sustained delayed enhancement - provides a useful radiological clue that should prompt consideration of HAS in the differential diagnosis of gastric malignancies, particularly when serum AFP is elevated. The spatial heterogeneity of enhancement within the tumor likely reflects its histological heterogeneity, suggesting that preoperative multiphasic CT may help identify tumors with significant hepatoid components.

### 4.2 Differential diagnosis: HAS vs. hepatocellular carcinoma

This constitutes the foremost diagnostic challenge. Our case and literature review highlight key discriminators, which are synthesized in **Table 1**. This task is complicated by the fact that hepatoid morphology and AFP production are not specific to gastric origin, with reported cases in diverse sites including the esophagus, colon, pancreas, lung, and urogenital tract (9–14). The presence of a dominant gastric primary, a non-cirrhotic liver background, and imaging patterns such as rim-enhancing liver lesions in the context of a gastric mass strongly favor metastatic HAS over primary HCC with gastric invasion, which is exceedingly rare.

### 4.3 Proposed conceptual diagnostic algorithm

The frequent misdiagnosis of HAS as HCC represents a critical clinical challenge with significant therapeutic implications. Based on our experience and literature synthesis, we propose a conceptual diagnostic algorithm intended as a clinical aid, not a validated tool (**Supplementary Table 2** provides a detailed stepwise guide). The algorithm emphasizes:

- Step 1: Initial Triage – For unexplained AFP elevation (>200 ng/mL), perform upper endoscopy with biopsy and multiphasic abdominal CT. Normal gastric mucosa on endoscopy does not exclude HAS; random biopsies should be considered if clinical suspicion remains.
- Step 2: Differential Diagnosis of Liver Lesions – When both gastric and hepatic lesions are present, use **Table 1** to distinguish metastatic HAS from primary HCC.
- Step 3: Multidisciplinary Confirmation and Treatment Planning – All suspected cases should be reviewed by a multidisciplinary team. When metastatic disease is present, determination of resectability should consider both the primary gastric tumor and any liver metastases, as well as technical feasibility and tumor biology.

### 4.4 Molecular pathogenesis: insights and hypotheses

Although no molecular analysis was performed in this patient, the aggressive behavior of HAS prompts consideration of potential pathways reported in the literature. These include aberrant activation of hepatic transcription factors (e.g., HNF4α, FOXA2), Wnt/β-catenin signaling, and liver-homing mechanisms (e.g., CXCR4, HGF/c-MET) (15–18). Future studies employing next-generation sequencing on larger cohorts are needed to validate these hypotheses and identify potential therapeutic targets.

### 4.5 Therapeutic considerations and surveillance

Complete surgical resection (R0) remains the cornerstone of potentially curative therapy, with long-term outcome studies supporting this approach (19). The benefit of perioperative chemotherapy is not well-defined for HAS; regimens are often adopted from gastric adenocarcinoma protocols (e.g., platinum/fluoropyrimidine). Given the high recurrence risk, intensive surveillance is warranted, including serum AFP monitoring monthly for 6 months then every 3 months for 2 years, and cross-sectional imaging (CT/MRI) every 3–4 months for the first 2 years (20, 21).

### 4.6 Strengths, limitations, and take-away lessons

#### 4.6.1 Strengths and limitations

Our report has several limitations inherent to its design as a single-center case report. First, the findings and the proposed diagnostic algorithm are derived from a single patient experience and require validation in larger, prospective cohorts. Second, the follow-up period (three months post-hepatectomy) is insufficient to assess long-term oncological outcomes and recurrence patterns of this aggressive disease. Third, the molecular pathogenesis discussions are speculative and based on literature review; future studies with next-generation sequencing are needed to confirm these hypotheses. Despite these limitations, the detailed quantitative imaging analysis and systematic diagnostic approach described herein provide valuable insights for clinicians encountering this challenging tumor.

#### 4.6.2 Take-away lessons

- AFP-producing HAS must be considered in the differential diagnosis of any hypervascular gastric tumor.
- Quantitative imaging can provide non-invasive clues to tumor biology.
- A systematic, multidisciplinary approach is essential for accurate diagnosis and optimal management of this aggressive disease.

## 5 Conclusion

This case of AFP-producing HAS, characterized by quantitative “hepatoid” enhancement on CT and managed through staged resection of primary and metachronous metastatic disease, underscores the pivotal role of integrated imaging-pathology correlation in overcoming diagnostic pitfalls. The following key insights emerge from our integrated approach:

- Quantitative imaging analysis can provide useful clues suggesting hepatoid differentiation.
- Conceptual diagnostic algorithms incorporating clinical, radiological, and pathological data are essential to distinguish HAS from HCC and other malignancies.
- Multidisciplinary management and aggressive surveillance are crucial given the tumor’s propensity for early metastasis and recurrence. Longer follow-up is needed to confirm durable outcomes.

Future efforts should focus on elucidating the molecular drivers of HAS to develop targeted therapies and validating imaging biomarkers that can predict biological behavior and treatment response.

## Data Availability

The original contributions presented in the study are included in the article/**Supplementary Material**. Further inquiries can be directed to the corresponding author.

## Glossary

AFP: Alpha-fetoprotein
HAS: Hepatoid adenocarcinoma of the stomach
HCC: Hepatocellular carcinoma
CT: Computed tomography
IHC: Immunohistochemistry
H&E: Hematoxylin and eosin
CEA: Carcinoembryonic antigen
HU: Hounsfield units
MRI: Magnetic resonance imaging
ROI: Regions of interest
R0: Complete resection (no residual tumor)
