# Supplementary material for: Case Report: AFP-producing gastric hepatoid adenocarcinoma with multiple liver metastases – integrating quantitative imaging and diagnostic decision analysis
Source: Front Oncol. 2026 Mar 25;16:1768282. doi: 10.3389/fonc.2026.1768282 (PMC13057322; doi:10.3389/fonc.2026.1768282)
Supplement: Supplementary file 1 [file Table1.docx]

**Supplementary Table 1. Timeline of Diagnostic and Therapeutic Interventions.**

| **Date/Time Period** | **Key Event** |
| --- | --- |
| Initial Presentation (February 2020) | Presented with dizziness and fatigue; markedly elevated serum AFP (918.88 ng/mL); CT revealed a gastric antral mass. |
| February 2020 | Gastroscopic biopsy performed, confirming the diagnosis of AFP-producing hepatoid adenocarcinoma of the stomach (HAS). |
| March 2020 | Underwent curative-intent Billroth II gastrectomy with D2 lymphadenectomy. |
| 4 months postoperatively (February 2021) | Surveillance CT identified multiple new hypervascular metastases in the right hepatic lobe. |
| March 2021 | Underwent right hemihepatectomy. |
| From April 2021 | Received adjuvant chemoradiotherapy (capecitabine with concurrent radiotherapy). |

**Supplementary Table 2. Diagnostic and clinical management pathway for patients with significant unexplained alpha-fetoprotein (AFP) elevation.**

| Step | Decision Point / Action | Branching Options | Next Actions / Explanatory Notes |
| --- | --- | --- | --- |
| 1 | **Initial Comprehensive Evaluation** | (Mandatory Workup) | 1. Detailed history and physical examination. 2. Multiphasic abdominal CT or MRI. 3. Upper endoscopy (EGD). |
| 2 | **Stratification Based on Imaging/Endoscopy Findings** | A: Suspicious gastric lesion identified | Proceed to Step 3. |
|  |  | B: No gastric lesion identified | Proceed to Step 8. |
| 3 | **Endoscopic Targeted Biopsy & Pathology** | (Perform Procedure) | Obtain targeted biopsies from the gastric lesion for histopathological examination. |
| 4 | **Interpretation of Pathological Subtype** | A: Conventional gastric adenocarcinoma | Manage according to stage-appropriate guidelines for gastric cancer. |
|  |  | B: Poorly differentiated carcinoma / Suspected hepatoid features | Proceed to Step 5. |
| 5 | **Hepatoid Adenocarcinoma (HAS)-Specific IHC** | (Perform Test) | Perform IHC panel including AFP, HepPar-1, CD10, CD34, etc. |
| 6 | **Interpretation of IHC Results** | A: IHC profile supports HAS | Confirm diagnosis of HAS. Proceed to Step 7. |
|  |  | B: IHC profile does not support HAS | Reassess diagnosis. Consider other poorly differentiated carcinomas. |
| 7 | **Staging and Management after HAS Diagnosis** | i. Complete Radiographic Staging | Perform CT of chest/abdomen/pelvis. Consider PET-CT if indicated. |
|  |  | ii. Assess for Liver Metastases | No liver metastases: Perform radical gastrectomy with D2 lymphadenectomy. Liver metastases present: Assess resectability (number, location, technical feasibility, future liver remnant, patient performance status). |
|  |  | iii. MDT Evaluation & Treatment Planning | Resectable (Oligometastatic): MDT discussion followed by combined or staged resection if feasible. Unresectable (Disseminated): Initiate systemic therapy, consider clinical trials, integrate palliative care. |
| 8 | **Management in the Absence of a Gastric Lesion** | A: High clinical suspicion persists | Perform EGD with systematic random biopsies (antrum, body, fundus). If positive, return to Step 4. |
|  |  | B: Low clinical suspicion | Proceed to Step 9. |
| 9 | **Systematic Workup for Alternative AFP Sources** | (Perform Investigations) | 1. Liver primary (HCC): Liver-specific MRI ± biopsy. 2. Pancreas/Biliary tract: MRCP, EUS. 3. Germ cell tumors: Testicular ultrasound, serum hCG. 4. Non-neoplastic causes: Evaluate for chronic liver disease. |
| 10 | **Management Based on Workup Results** | A: Alternative diagnosis established | Direct therapy towards the identified diagnosis. |
|  |  | B:  No alternative diagnosis found | Enter monitoring phase: Measure serum AFP every 1-3 months, repeat imaging in 3-6 months. Consider repeat EGD if AFP rises. |
| 11 | **Structured Surveillance Protocol** | (Standardized Follow-up) | For all managed patients:  • Clinical assessment: Every 3-6 months.  • Serum AFP: Monthly for 6 months, then every 3 months for 2 years.  • Cross-sectional imaging (CT/MRI): Every 4-6 months for 2 years, then annually.  • Endoscopy: Annual EGD if indicated. |

Abbreviations: AFP, alpha-fetoprotein; CT, computed tomography; EGD, esophagogastroduodenoscopy; HAS, hepatoid adenocarcinoma of the stomach; HCC, hepatocellular carcinoma; IHC, immunohistochemistry; MDT, multidisciplinary team; MRI, magnetic resonance imaging.
